# Copy of a Letter from Dr. Friese to Mr. Ring, Dated Breslaw, June the 9th, 1805

**Published:** 1805-09-01

**Authors:** D. Friese


*Copy of a Letter from* Dr. Friese *to* Mr. Ring, *dated Breslaw, June the 9th,* 1805.

Dear Sir,

THE unremitting zeal with which you have endeavoured to promote the Jennerian discovery in your country, and the interest you have so philanthropically shewn, on hearing of its first providential introduction into Silesia, will, I hope, excuse me, when I take the liberty to trouble you with some further account of the successful progress which that invaluable prophylactic has since made in this part of the Prussian dominions. Should the following Report be deemed acceptable to the Editors of the Medical and Physical Journal, I shall feel myself highly gratified, by adding, as a foreigner, some further proofs to the evidence, that vaccination, when properly managed, every where proves a permanent security against the small-pox

I could not but be astonished when I read over the pamphlets of Messrs. Goldson and Squirrel. I apprehend the alarm they excite, will come at too late a period for them to flatter themselves with much success. At any rate, I am convinced the new doctrine which they promulgate, will find but few proselytes in Germany; where both the government and the people are more and more sensible of the advantages of the new practice; and where similar equivocal arguments, advanced some years ago by the late Dr. Herz, Mr. Ehrman of Frankfort, and Dr. Mattreschka of Prague, have been silenced by time and experience.

You remember, perhaps, by my former letter, that there was also an adversary of some celebrity in Silesia, who rose up against the vaccine inoculation, at its first introduction into this country. His name is Mogallæ, a physician known in Germany by his very valuable writings on the several mineral waters, and bathing places of Silesia, and by some other works on the veterinary art; but I have the pleasure to inform you, that this respectable practitioner has been converted by reason and evidence into one of the warmest friends and promoters of vaccination. I must add, that it was particularly by his assistance, that we are now in possession of two public vaccine institutions at Breslaw and Glogaw; which are to be regarded as the centres from which the practice is spread, and continues to be spread, through every quarter of the province.

His Majesty has been graciously pleased to appoint me not not only a counsellor of the medical department of Silesia, and a director of the royal institution at Breslaw; but he has also honoured me with the superintendancy of this new branch of the healing art, in the department of the royal chamber in this capital. The establishment of the vaccine institution has been so expeditious, that I found myself enabled to begin my operations on the 14th of April 1804, with equine matter, sent to me by my friend Dr. De Carro; from the very same source of which you have spoken in the Medical and Physical Journal for November 1804.

I have the pleasure to subjoin a statement of the number of persons inoculated at the Royal Institution of Breslaw, from its establishment till the present day; as well as a general abstract of vaccinations performed by different medical men in all the subordinate districts of the department of Breslaw during the year 1804, from the annual reports. I hope you will see by these lists, that the progress of the Jennerian inoculation, during the last year, has, by far, surpassed those of all preceding, since the year 1800. I do not yet know all the particulars of the results of vaccination in the second department of the Royal Chamber at Glogaw; but I am informed by private letters, that the number of persons vaccinated there is more than ten thousand; the total number of inoculations successfully performed in the last year, amounting to nearly thirty-four thousand; besides some thousands more, who have been vaccinated by surgeons of the army.

Government have pursued measures well calculated to promote vaccination. I have been charged with the commission of writing not only a popular publication on it, which is ordered to be printed, and distributed among all classes of people; but also a brief instruction for the physicians and surgeons of the province; wherein I have endeavoured to give an account of this new discovery; and also to acquaint them with the genuine and spurious pustule, and the best method of inoculating and treating the disease.

Another measure, not less favourable for promoting uninterrupted vaccination at the central institution of this city, is the grant of a sum of two hundred rix-dollars annually, destined for small premiums at a dollar each, to be distributed among children of the lower classes; who, for the sake of such a trifle, willingly comply with the rules and conditions of the inoculators. It must be particularly ascribed to this encouragement, that the institution tion has been enabled constantly to provide, not only all Silesian inoculators, but also several of those of the adjacent countries, with fresh and genuine cow-pock matter, having disseminated during the last year 1312 armed ivory lancets of Dr. De Carro's invention.

Our mutual friend of Vienna has informed you, that several German clergymen have participated the labour of promulgating the new practice; and I have the pleasure to acquaint you, that there are many in this country likewise, who partake in this laudable design. I even venture to assert, that there are some country clergymen in our province, who are so well acquainted with vaccination, both in theory and practice, and have conducted it with so much care and skill, as justly to deserve the name of benefactors of their parishioners. In order to acknowledge the merits of these respectable divines, and at the same time to excite a laudable emulation among all the medical men of the province, government have granted to several of those who have distinguished themselves in this line, small premiums from thirty to fifty rix-dollars.

I find by the Medical and Physical Journal for March 1805, that Mr. Goldson is indefatigable in promulgating his cases of small-pox subsequent to vaccination; having just published a second Treatise on that subject. I have perused the last numbers of this before mentioned Journal, as well as several other reflections written on this occasion. I wonder that there are people who think the punishment you and Dr. Walker have inflicted on Mr. Goldson, in your answer to his productions, too severe; nay, that there are some anonymous writers, who pretend Mr. Goldson's pamphlet is entitled to the most serious attention of the faculty. For my own part I cannot find, that the evidence of cases related by him, in order to prove that vaccination affords no permanent security against the infection of the small-pox, is so clear and satisfactory as they pretend. I shall have an opportunity of communicating to you some similar cases, which happened in Silesia; but I assure you, that after due inquiry, this could not in the least degree alarm the public, who, on the contrary, have every day had the satisfaction of seeing, that the cow-pock is the only powerful and permanent preventive of that dreadful scourge of mankind, the small-pox; which, in the course of the last year, destroyed several thousands of the rising generation, while those who had regularly undergone the operation, remained secure from its malign influence and its dreadful contagion.

Few

Few mistakes indeed have lately been committed here, in the practice of vaccination. I shall mention one which occurred in the year 1802, at Brieg, a city, six German miles from this place. Mr. Taber, a surgeon, inoculated several children from the arm of a child, in whom the pustules were already approaching to the scabbing process. He confesses, he had at that time never seen the process of a genuine pustule. This occasioned him to mistake the ulcers produced in the arms of these children, for the true kind; and to transfer from them a spurious and purulent matter to thirty-one persons, thinking they would all be perfectly secured by this operation. The small-pox, however, making its appearance at Brieg the next summer, three of the children were attacked by the disorder. At length perceiving his error, and having in the mean time acquired a sufficient knowledge of the true progress of vaccination, he inoculated the rest of his patients with genuine matter; in consequence of this, they all took the disease in the regular way, and resisted the small-pox; which then committed great ravages among those children, whose parents had refused the benefit of vaccination.

I cannot omit stating three other cases of supposed vaccination, in children of one family, in a village in this neighbourhood; which greatly resemble those in Fullwood's Rents, and at Kensington. One child, a girl of four years, was attacked four weeks after by the confluent small-pox. I saw her on the 11th day of the disease; and, as the physician who had performed the inoculation, and accompanied me, confessed he had not had an opportunity of observing its progress, I immediately tried a second vaccination on the two remaining boys, who had at this time large greenish and irregular scabs on their arms, remaining from the first operation. The inoculation succeeded, and on the 6th day two flat vesicles appeared, with the common depression in the centre; but on the 7th day, they both became feverish, and on the 9th a small crop of distinct variolous eruptions broke out. The father, though a common country labourer, reproached himself for having delayed a second vaccination of his children; who, by rubbing and scratching the pustules, had entirely destroyed the progress of the first.

Accept my best thanks for the honour you have done me, in the second Volume of your excellent work; and believe me to be, With great respect,

Dear Sir,

Your obedient humble Servant,

D. FRIESE.

